# New Insight on Biological Interaction Analysis: New Nanocrystalline Mixed Metal Oxide SPME Fiber for GC-FID Analysis of BTEX and Its Application in Human Hemoglobin-Benzene Interaction Studies

**DOI:** 10.1371/journal.pone.0102992

**Published:** 2014-07-28

**Authors:** Reza Hosseinzadeh, Ali Akbar Moosavi Movahedi, Hedayatollah Ghourchian

**Affiliations:** 1 Institute of Biochemistry & Biophysics (IBB), University of Tehran, Tehran, Iran; 2 Chromatography Research Group, Academic Center for Education, Culture and Research (ACECR), Urmia Branch, University of Urmia, Urmia, Iran; 3 Center of Excellence in Biothermodymanics, University of Tehran, Tehran, Iran; Tsinghua University, China

## Abstract

Nanocrystalline mixed metal oxides (MMO) of various metal cations were synthesized and were used for coating a piece of copper wire as a new high sensitive solid phase micro extraction (SPME) fiber in extraction and determination of BTEX compounds from the headspace of aqueous samples prior to GC-FID analysis. Under optimum extraction conditions, the proposed fiber exhibited low detection limits, and quantification limits, good reproducibility, simple and fast preparation method, high fiber capacity and high thermal and mechanical durability. These are some of the most important advantages of the new fiber. The proposed fiber was used for human hemoglobin upon interaction with benzene. Binding isotherm, Scatchard and Klotz logarithmic plots were constructed using HS-SPME-GC data, accurately. The obtained binding isotherm analyzed using Hill method. The Hill parameters have been obtained by calculating saturation parameter from the ratio of measured chromatographic peak areas in the presence and absence of hemoglobin. In this interaction, Hill coefficient and Hill constant determined as (n_H_ = 6.14 and log K_H_ = 6.47) respectively. These results reveal the cooperativity of hemoglobin upon interaction with benzene.

## Introduction

Small chain alkylbenzenes are a class of toxic volatile organic compounds such as benzene, toluene, ethylbenzene, and xylenes, commonly known as “BTEX”. The BTEX compounds represent some of the most hazardous components of gasoline. The BTEX compounds have been more frequently associated with risk to humans than with risk to non-human species such as fish and wildlife. It is limited because only a very small amount of these compounds are absorbed by plants, fish, and birds and because these compounds are volatile and tends to evaporate into the atmosphere rather than remaining in surface waters or soils [1]. However, volatiles such as BTEX compounds can pose a drinking water hazard when they accumulate in ground water. These compounds are an important class of volatile environmental contaminants and are frequently analyzed in environmental and drinking waters. Nowadays, with the development of human societies and industries, entry of pollutants in the environment has increased dramatically, leading to many new diseases that they can be called industrial diseases. Widespread increasing of poly aromatic hydrocarbons in human environment and air, water and soil contamination of the BTEX hazardous compounds, especially benzene induced several new diseases. Chronic effects of BTEX include damage on the liver and harmful effects on the kidneys, heart, lungs, and nervous system. Also, aromatic hydrocarbons have long been associated with the induction of non-immune hemolytic anemia or met-hemoglobinemia. In particular, episodes of non-immune hemolytic anemia have been observed in industrial workers exposed to the organic solvents benzene and toluene [2]–[4]. Although BTEX compounds such as toluene and xylenes are not officially recognized as part of the active ingredients of the pesticide containing it and are therefore part of the so-called "inert" these compounds are nevertheless far from "safe" at all concentrations to all life forms [1]. Study of the effect of these compounds on the biological macromolecules and cells can be helpful in understanding of the mechanism and pathway of hazard effect induced by BTEX compounds for considering suitable medical purposes. Various methods have been used to study the interaction of small molecules to proteins including equilibrium dialysis, potentiometery, FTIR, ultrafiltration and gel filtration [5]–[9]. These techniques are usually time consuming, can suffer loss of the analyte to membranes and can create a shift in the binding equilibrium during separation and are limited in some cases of ligands especially for volatile organic compounds due to specific characteristic of these compounds. Solid phase microextraction can used for these studies, accurately. Solid phase microextraction (SPME) was initially developed as a simple extraction method originally introduced by Pawliszyn and co-workers in the early 1990s [10]. Now a day's applications for SPME have extended from environmental analysis [11]–[14] various foods [15]–[18], chemicals [19]–[22], forensic and pharmaceutical and also are extended in vivo or in situ analysis and diagnostic medical or biological applications [23]–[26]. One of interesting application is that SPME can be used in negligible-depletion extraction mode for free analyte determination in matrix samples. Therapeutic drug monitoring (TDM) is important in effective dosage determination for everybody in this regards SPME can be very useful for determining free and bond fraction of drugs in the blood or body fluids by considering proteins and macromolecules binding affinity to the certain drugs. The SPME can be applied by exposing the fiber in the headspace above the sample (headspace SPME) or direct immersion in the sample solution. The advantage of headspace SPME sampling is that the matrix in the sample cannot interfere with the fiber.

The equilibrium between the free and the protein bound analyte is not disturbed. So, the extracted amount of ligand is proportional to the free ligand concentration without interference from the protein binding matrix [9].

Due to the simplicity, sensitivity, re-reducibility, fast analysis, low cost and ease of preparation of SPME fibers a new nanocrystalline mixed metal oxide of Ce-Cu-Zr was synthesis and used on construction of a new laboratory made SPME fiber in this work.

The proposed fiber was also successfully applied to study Benzene interaction with human hemoglobin (h-Hb) and quantification of BTEX compounds in water samples.

## Experimental

### 2.1. Materials

Benzene, toluene, ethylbenzene, potassium sulfate were all from E. Merck (Germany). Xylene isomers purchased from FLUKA (Switzerland). Acetone and THF were supplied from E. Merck. Copper (II) nitrate tri-hydrate, cerium (III) nitrate hexa-hydrate, zirconyl chloride octa-hydrate, PVC powder was purchased from Sigma and is in analytical grade. Copper wire (0.35 mm o.d.) was supplied locally and used after cleaning. Nitrogen and hydrogen gases (99.999%purity) were from Air Products (UK). All the solutions were prepared with doubly distilled water.

#### Synthesis of nanocrystallin MMO (X-Ce-Zr)

**Figure 1:**
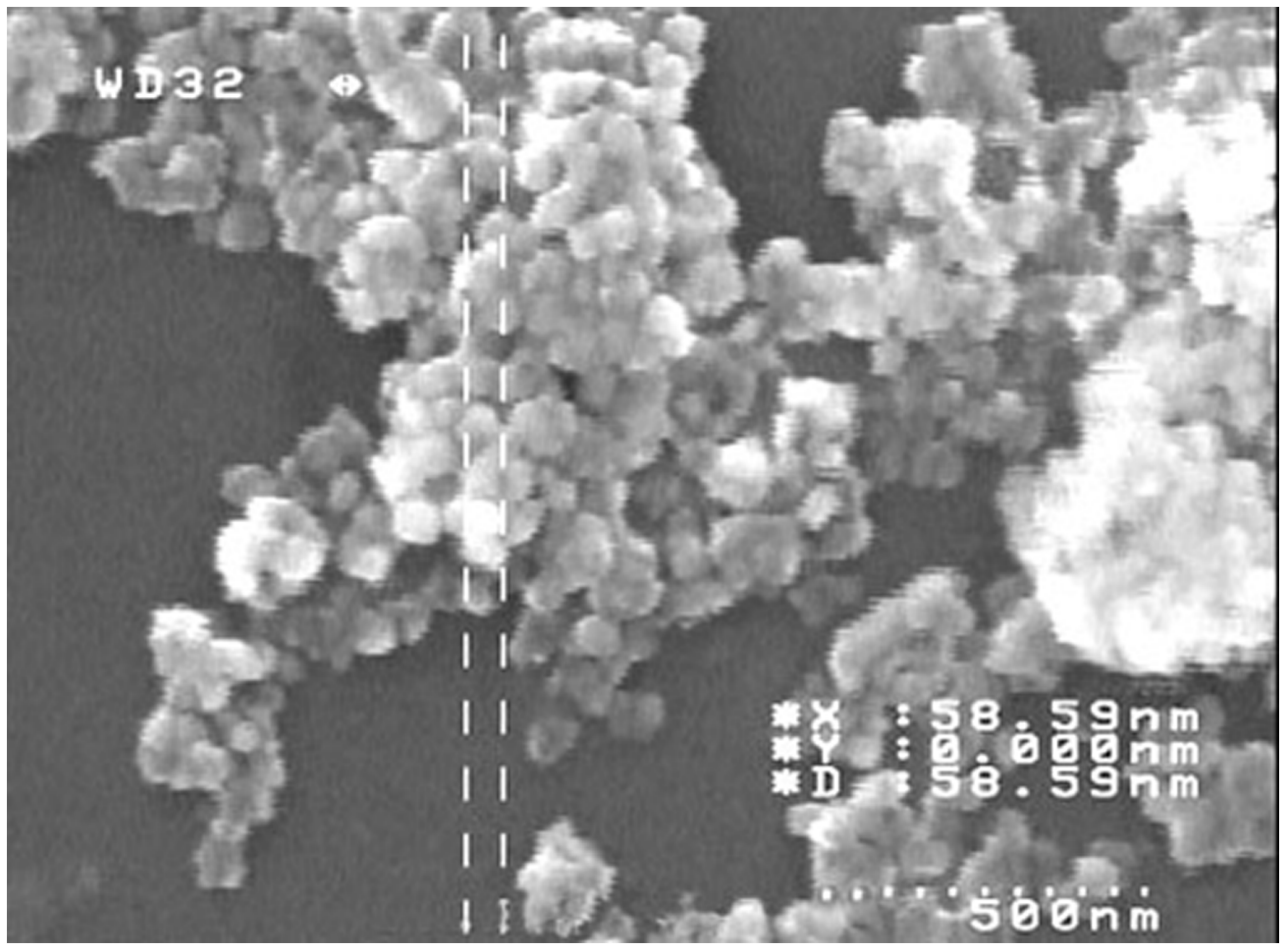
FE-SEM image of synthesized crystalline nanoparticles of Ce-Cu-Zr mixed oxides.

The MMO nanocrystallin particles of various mixed metal solutions (X =  Cu, Ni, Mn, Zn, Co) were synthesized by drop wise addition of an aqueous solution of sodium hydroxide into a stirred mixed aqueous solution of X, Ce and Zr solutions (containing appropriate amounts of each cation) in a beaker, up to pH 10 [27]. The resulting precipitate was left stirred for 6 h for further aging of precipitate. After complementation of precipitation process, the sediment was washed for several times and then dried at 120°C overnight, followed by calcinations at 850°C for 6 h in air atmosphere. SEM images of synthesized nanoparticles are presented in Figure 1.

#### Human adult hemoglobin extraction

Human hemoglobin was extracted in our laboratory using the method was described previously [28]. In brief: new, fresh and heparinized blood was centrifuged at 3000 rpm to remove plasma components. The upper yellowish solution was decanted and the packed red cells were washed three times in an isotonic saline solution (0.9% NaCl) in the ratio of 1∶10 for 5 min and subsequently centrifuged at 10000 rpm for three time. Red cells were osmetically lysed with cold double distilled water. Membrane components were removed by centrifugation (10000 rpm). The soluble Hb was centrifuged at least two additional times at high speed to remove any insoluble materials (18000 rpm). The hemoglobin solution was then brought to 20% saturation with ammonium sulfate, left standing for about 20 min, and centrifuged at 20000. The resulting Hb solution was dialyzed 3 times for 24 h against 0.2 M phosphate buffer solution (pH 7.4).

#### Ethics statement

1- Board of Education and Research in Institute of Biochemistry and Biophysics (IBB), University of Tehran.

2- The satisfying of students regarding blooding is verbal.

3- Just the blooding from the satisfying students (at least 10 ml.) is permitted under the medical permission. Blooding was performed by medical doctor.

### 2.2. Methods

#### Preparation of SPME fiber

The synthesized nanocrystallin powder was used for SPME fiber preparation. 0.06 g of Ce-Cu-Zr nanocrystallin powder was mixed with 0.02 g PVC powder and then 5 mL THF was added and well mixed (the optimized mixture as mentioned in next step). After a few minutes, THF was evaporated until a viscose suspension was formed. A piece of copper wire (2 cm), which was assembled on the laboratory made SPME devise, was cleaned carefully using acetone and coated by the prepared homogeneous suspension. After evaporation of THF, a layer of sorbent was coat on a copper wire. Conditioning of the prepared fiber was done in an injection port of a gas chromatograph at 280°C for 10 min to remove any fiber contaminations and sedimentation.

#### Coating composition, thickness and fiber length

The amount of analyte adsorption on the fiber depends on the thickness, length and composition of coating materials and on the distribution constant for the analyte between headspace and coating. Fiber coating material composition was optimized based on maximum extraction efficiency in the adsorption of analytes. Various composition was considered and examined based on weight percent of nanocrystalline adsorbent (MMO) and PVC as: 90∶10, 75∶25, 50∶50, 25∶75 and 10∶90. The fibers were prepared based on these percentages of nanocrystalline compound and used in SPME determination of analytes. Based on maximum analytes extraction efficiency, the 75∶25 composition was considered as optimum coating composition. Generally thick coating is suitable for volatile compounds, in the other words thicker coating is used to retain volatile compounds and transfer them to the GC injector without loss of analyte. Thin coating is most effective for semi-volatile analytes and is used to ensure fast diffusion and release of higher boiling compounds (semi-volatile analytes) during thermal desorption. However much more thicknesses is not suitable for analysis because of tailing effect in chromatograms and analytes peaks and decreasing peak separation and determination also desorption of analytes in thick coatings need more time and injector temperature that decreased shelf life of fiber and make thermal decomposition of coating materials. In here we considered ∼40 µm thickness for our study. Fiber performance as a function of extracting phase volume, was examined based on various fiber length. 1, 1.5 and 2 cm length are used. According to results 2 cm was considered for other studies as optimum fiber length. It must be mentioned that the use of longer fibers in SPME is uncommon and instrumental limitation must be considered, so 2 cm was selected as optimum length.

#### Standards solution preparation and microextraction procedure

Stock solution of the mixture of standards was prepared as below: 2 mL of each analyte thoroughly added to a closed vial, well mixed and stored at 4°C. Aqueous solutions of BTEX compounds were made by dilution of appropriate volumes of stock solution with water in calibration flasks. To a sample vials, appropriate volume of salt solution (optimized concentration of salt) was placed in and spiked with known amounts of the analytes then vial was sealed and its temperature was adjusted to experimental temperatures. The sample solution was stirred and the SPME fiber was inserted into the headspace of the sample solution and left for optimum time. After analytes adsorption and extraction, the fiber was removed from the vial and immediately inserted into the hot injection port of the GC at 220°C (optimum desorption temperature) 50 seconds to desorption the analytes in GC glass liner.

### 2.3. Instruments and apparatus

A Shimazdu gas chromatograph (Shimadzu GC-15A, Japan), equipped with a FID detector, split/split-less injector was used with an OV-101 capillary column (50 m×0.25 mm I.D.). Shimadzu solution software was used for data processing. Magnetic stirrer (ZAG Shimi, Iran) was used for sample agitation. A homemade SPME device was used. 10, 25 and 50 mL glassy vials were used for head space SPME procedure.

#### GC conditions

Temperature program for GC separations of analytes was examined and considered as mentioned in here. The initial column oven temperature was adjusted at 55°C for 7 min and then raised at 20°C/min to 100°C and held for 2 min after that by 40°C/min temperature ramp increased to 175°C and held for 7 min. High pure nitrogen gas was used as carrier and makeup gas. The temperatures of the injection port and detector were set at 220 and 280°C, respectively. Injections of analytes were made in split mode by 1∶10 split ratio.

## Results and Discussion

### 3.1. Coating fiber parameters

#### Optimization of fiber coating composition

To achieve the best composition and suitable material for fiber coating, the synthesized compounds were examined and the optimum percentage of blended fiber materials was selected. The amount of analyte adsorbed by the fiber depends on the distribution constant for the analyte between headspace and fiber. The fiber material with high potential to analytes can shift distribution equilibrium to fiber adsorption due to Le Chatelier's principle and can affected limit of detection for determination procedure. Hydrophobicity and polarity of fiber coating can be optimized based on analyte hydrophobicity and polarity based on optimization of fiber coating materials. According to the results obtained using extraction method, 75∶25 (MMO: PVC) mixture of coating materials exhibited the maximum extraction efficiency compared with the other compositions for extraction of analyte compounds and was selected as the perfect composition for other optimization studies.

#### Temperature and time optimization of fiber microextraction

**Figure 2:**
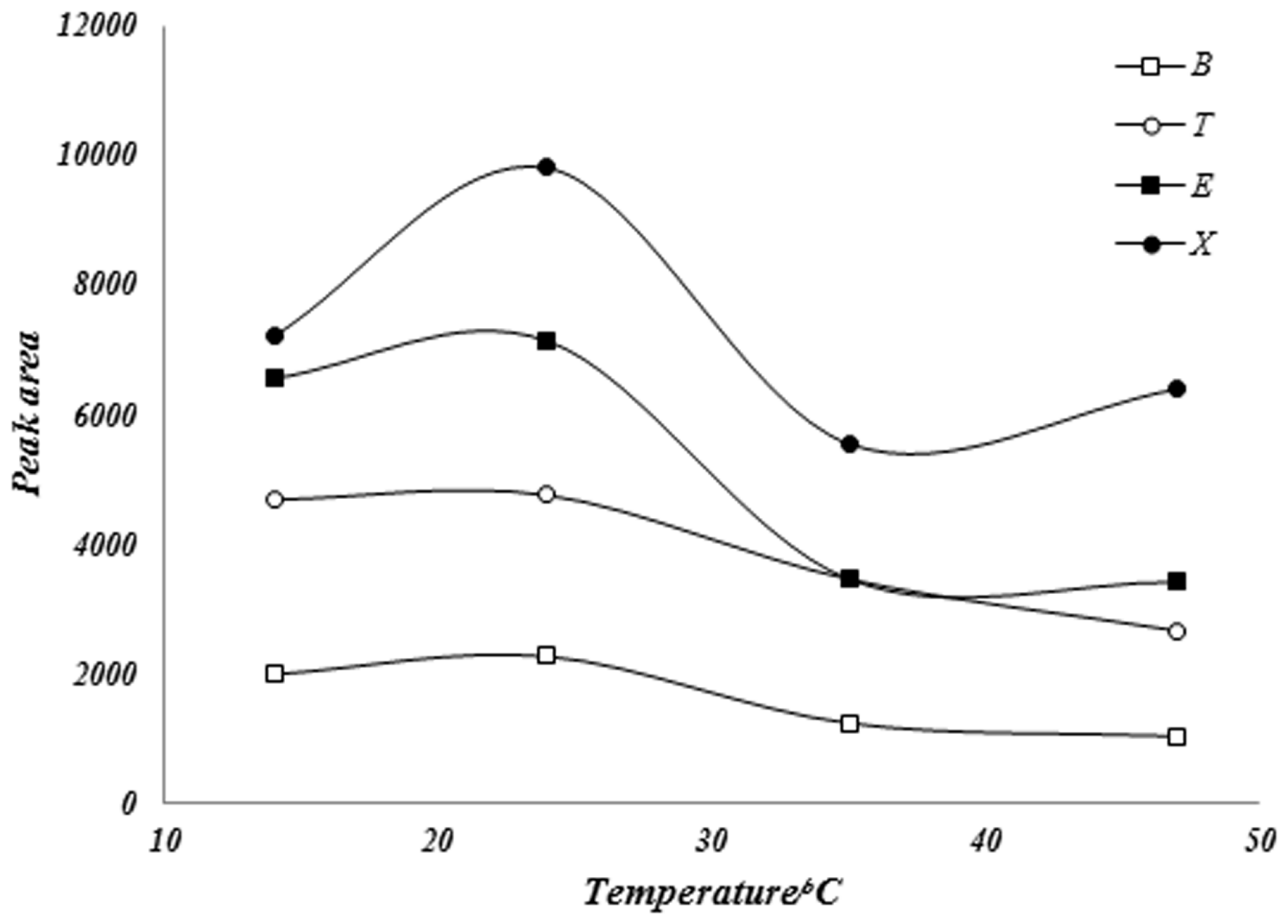
Sample solution heating effect on BTEX adsorption from head space phase into the fiber.

In order to find the best temperature and time profile for extracting the BTEX compounds, the effect of these parameters in the extraction of the analytes were tested. Figure 2 reports the results obtained with three experiments with standard solution of BTEX compounds to compare the effect of four different temperatures: 14, 25, 35 and 47°C, in the extraction yield. Extraction temperature has a dual effect, increase of temperature caused an increase in volatility of the analytes, but high temperature decreases the amount of analytes adsorbed onto the fiber due to this fact that analytes adsorption on fiber commonly are physical adsorption and due to the weak and physical interactions analytes molecules are adsorbed on the fiber and by further increasing in the extraction temperature these weak bindings disconnect. The best results were obtained for an extraction temperature of 25°C.

The optimum time for extraction should be the time of equilibrium reaches, since the mechanism of extraction is based on the equilibrium between analyte and the coated materials of the fiber. The exposure time of analytes distribution equilibrium between the fiber and the headspace phase is one of the most important parameters in SPME extractions. This optimization test was done in the range of 0–25 min.

**Figure 3:**
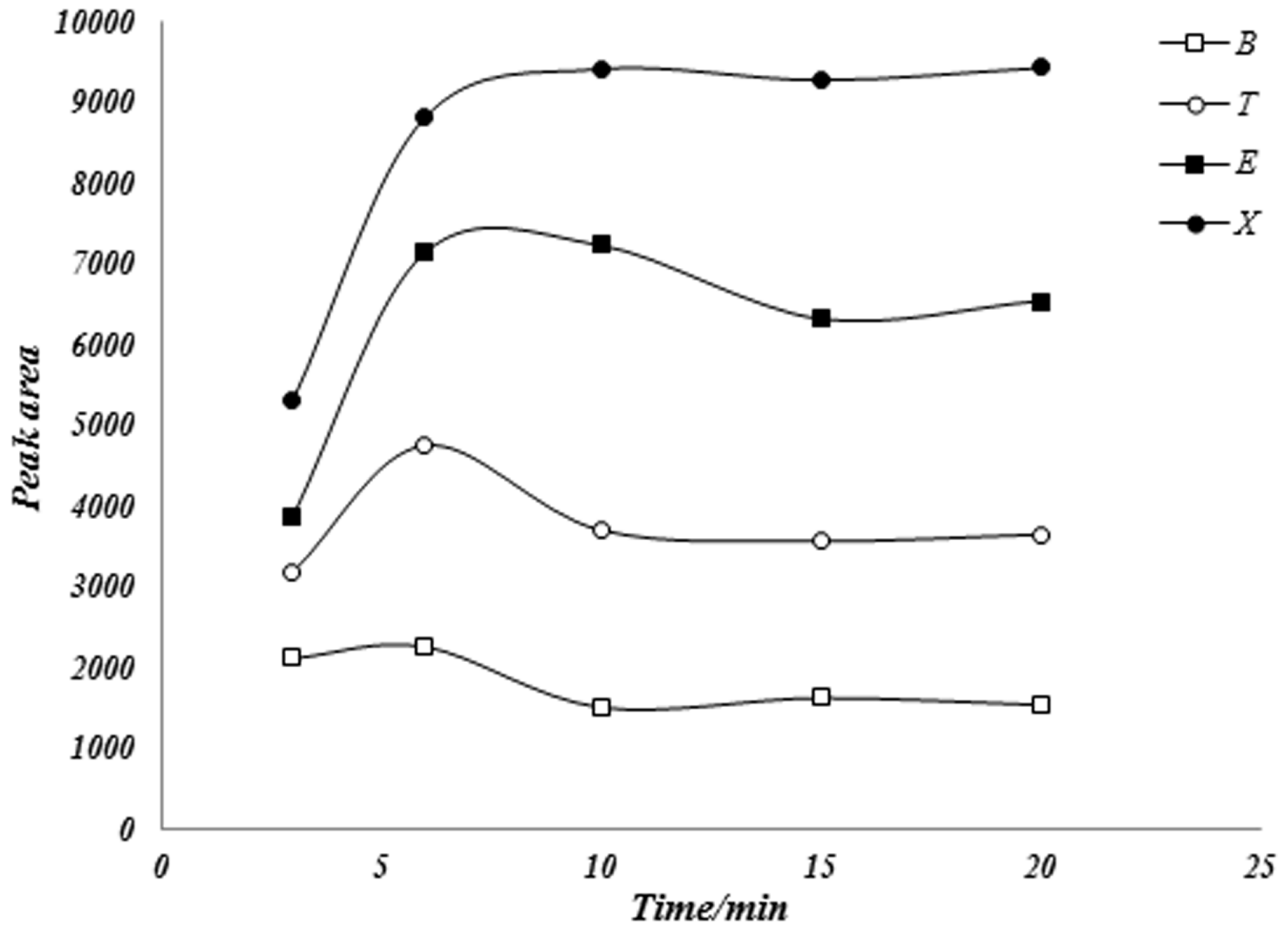
HS-SPME adsorption-time profile for BTEX into proposed fiber.

Extraction times were examined and compared at optimum temperature using the Ce-Cu-Zr fiber in the headspace-sampling mode. Figure 3 shows the influence of the time in the extraction procedure. It is clear that the extraction time profile depends on the individual analyte. In the consider time range, BTEX compounds reached the optimum equilibrium extraction in less than 8 min. According to Figure 3, and considering a compromise between the duration of the analysis and the time of the extraction, an extraction time of 6 min for samples was selected for subsequent analysis, because this time provides sufficient extraction yield of the analytes and after this time the extraction was not significantly improved.

#### Optimization of desorption time and temperature

**Figure 4:**
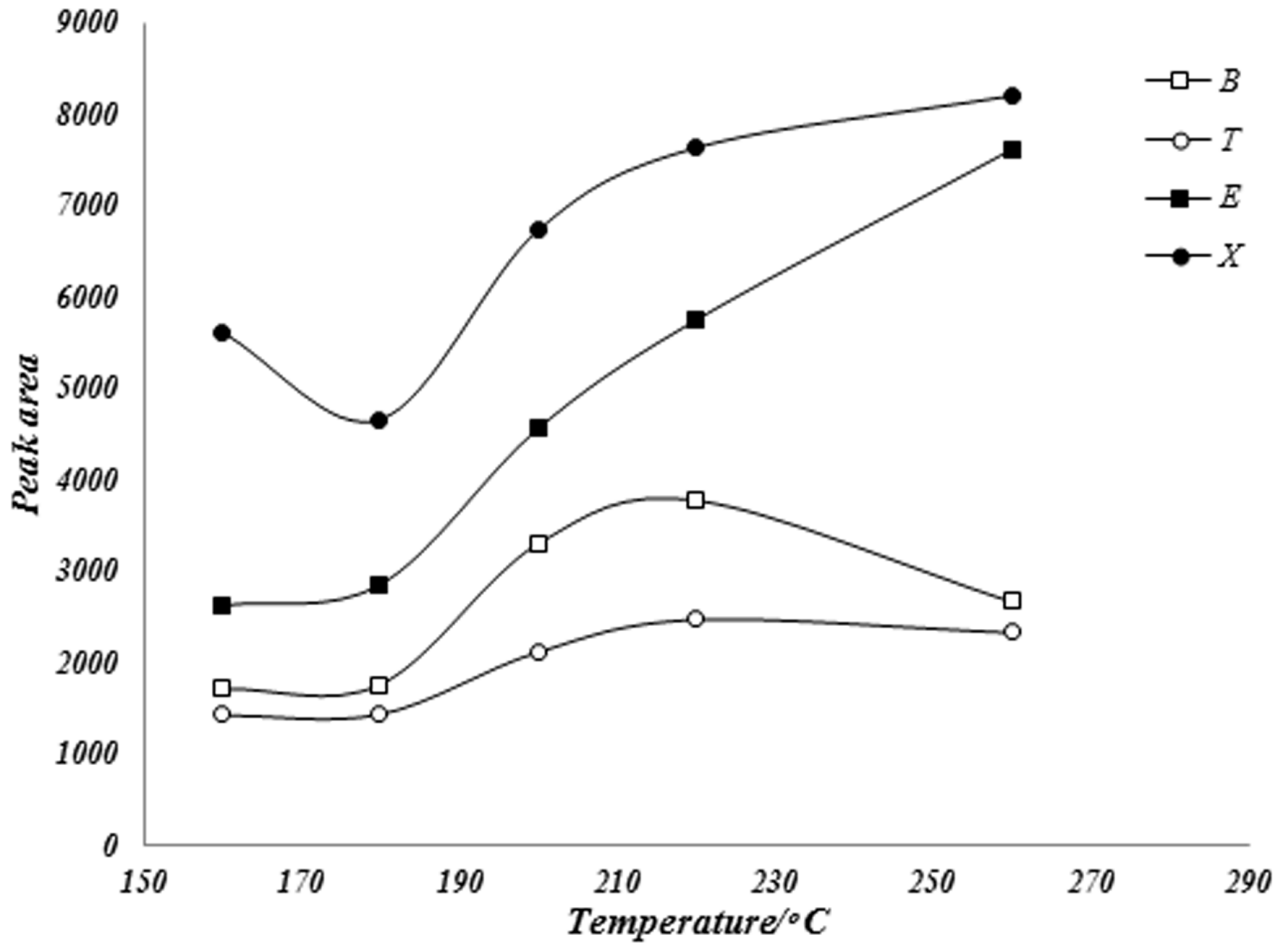
Effect of temperature in desorption of BTEX compounds from the fiber in GC injection port.

**Figure 5:**
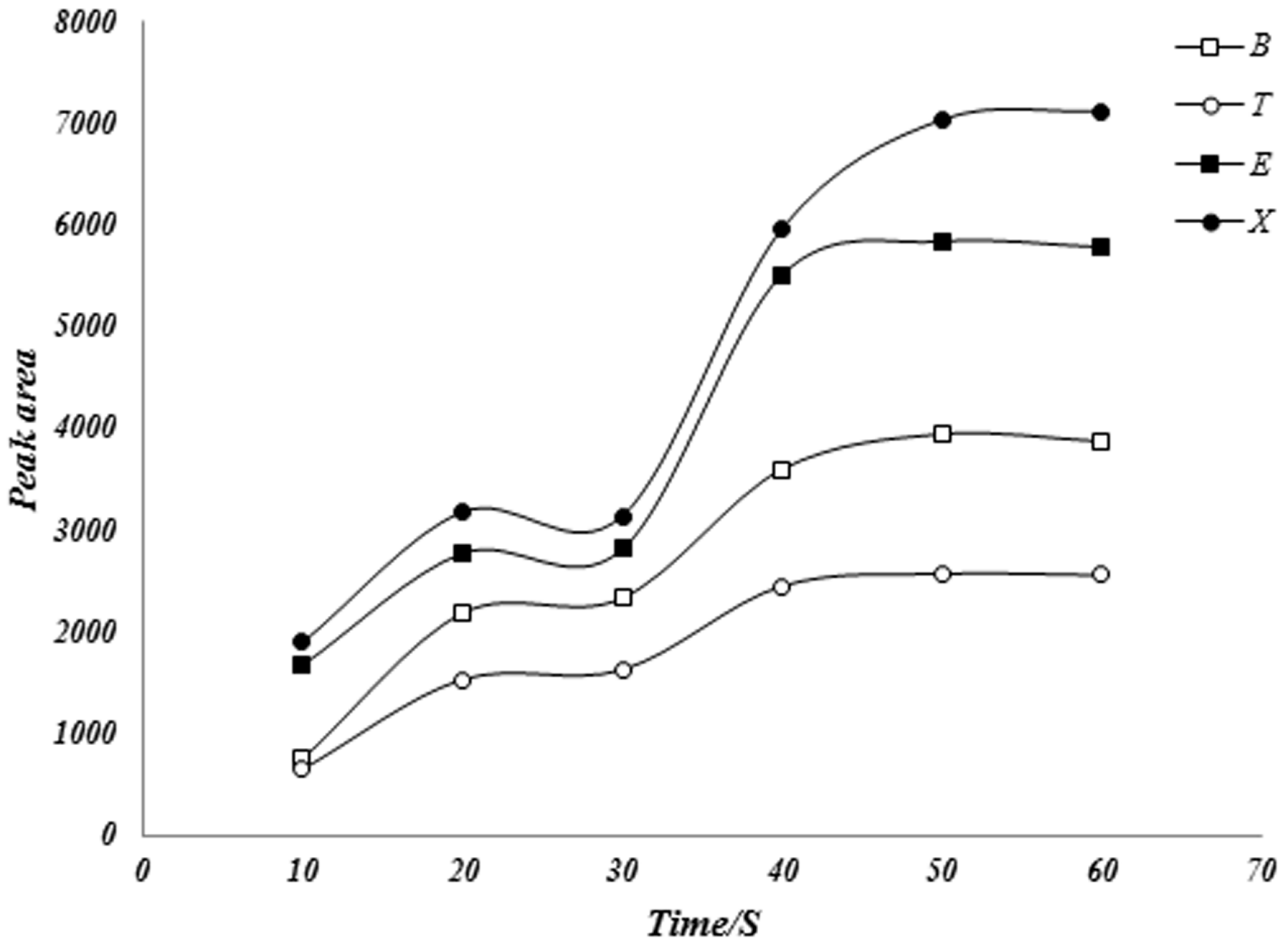
Effect of desorption time for the BTEX compounds by proposed SPME fiber.

Purpose of optimization of desorption time and temperature is typically to eliminate carry over and improve peak shape. Desorption parameters are selected such that any analyte remaining on the fiber after desorption will not cause variance in the results outside of normal method precision. The required desorption temperature may be close to the temperature of tolerance of the fiber coating, which may shorten the life of the fiber and decompose it. Incomplete desorption of analyte from fiber coating in the gas chromatograph injection port can be make two problems in measurements: 1) the obtained result in first injection is less than real quantity of analytes and 2) in next injections, obtained results for analytes can be more than real one due to slow release of analyte in next injections. So, analytes desorption is one of important steps in fiber optimization. One of great advantages of the proposed fiber is coating thermal stability (upper than 300°C). Due to this advantage, we can consider high desorption temperature for complete removing of analytes in injection port. Optimization of desorption temperature and time were done at various temperatures ranging from 160 to 260°C and various desorption time (10–60 seconds). Obtained results showed that suitable desorption was achieved for all the extracted analytes after 50 seconds of desorption at a temperature of 220°C (Figures 4 and 5).

For validation of the analytical method, limits of detection, linearity, precision and quantification of the method were estimated. Results are summarized in Table 1.

**Table 1:** Hill binding data and SPME performance and validation data for proposed fiber.

Compound	r^2^	LOD (ng.L^−1^)	*LOQ* (ng.L^−1^)	Hill (Hb-binding data)		
				n_H_	logK_H_	r^2^(Hill)
Benzene	0.998	1.93	5.76	6.14	6.47	0.996
Toluene	0.999	2.34	6.97	-	-	-
Ethylbenzene	0.999	0.66	2.12	-	-	-
Xylene	0.999	0.28	0.85	-	-	-

#### Effects of salt concentration on microextraction

**Figure 6:**
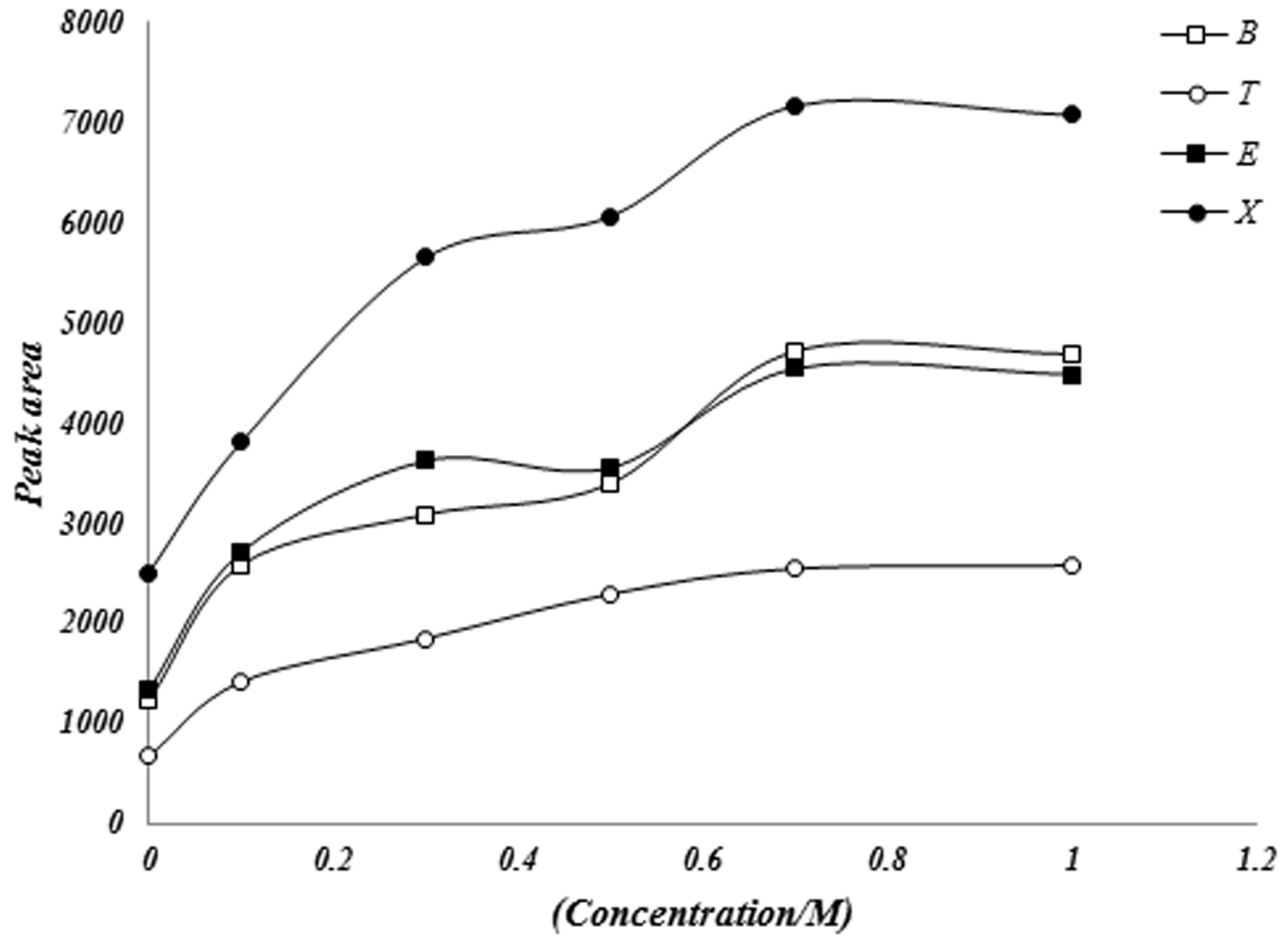
Effect of salt concentration on the determination of BTEX in sample by SPME GC-FID.

The suitability of the headspace SPME technique for the extraction compounds depends on the transfer of the analyte from sample gaseous phase and therefore to the fiber. This process can be affected by the ionic strength and presence of hydrophobic or hydrophilic compounds in sample solution. In the present work, the effect of potassium sulfate concentration (ranging from 0 to 1 mol.L^−1^) was investigated and the extraction efficiencies were monitored. The peak area increased with increasing salt concentration up to 0.7 mol.L^−1^ (Figure 6). In the presence of hemoglobin we used phosphate buffer solution (2 mM phosphate buffer solution at pH 7.4) for adjusting ionic strength and pH of solution in biological range.

### 3.2. Quantitative properties of the proposed fiber

After optimization of microextraction parameters, quantitative characteristics of the proposed fiber were determined. These included calibration curve correlation coefficients, limits of detection and quantifications that are summarized in table 1. There are several terms that have been used to define LOD and LOQ. Commonly, the LOD is define as the lowest concentration of an analyte in a sample that can be detected, but not necessarily quantified, under the stated conditions of the experiment. The LOQ is the lowest concentration of analyte in a solution that can be determined with suitable precision and accuracy in the experimental conditions. The LOD and LOQ parameters were estimated from signal- to-noise method. In this method, peak-to-peak noise around the analyte retention time is measured, and subsequently, the concentration of the analyte that would yield at least 3×(S/N) ratio is estimated for LOD parameter and 10×(S/N) ratio is acceptable for LOQ data. The high correlation coefficients (0.998–0.999), low detection and quantification limits, in the range of 0.28–1.93 ng.L^−1^ and 0.85–6.97 ng.L^−1^, respectively made the proposed method suitable for the quantification of the BTEX.

### 3.3. Study of method reliability, recovery and reproducibility

Relative standard deviations (RSD %) were estimated for five replicate measurements using a single fiber. The RSD values were less than 4.83% for all measuring analytes, which indicates that the proposed method is repeatable and data have good reliability. Also, three different fibers were consider for reproducibility studies and fiber-to-fiber relative standard deviations were calculated. RSD values are summarized in the table 2. It should be mentioned that the proposed fiber shows good thermal stability and durability and no sign of bleeding even at the high temperatures ∼300°C. High thermal stability of proposed fiber can provide complete desorption of extracted analytes by using a high injection temperature, that is another advantages of new fiber. Recovery of added analytes to water sample solution was considered for considering matrix effect on measurements. The recovery percentages more than 97% indicate that the method can be used for analytes determination in aqueous solution accurately.

**Table 2:** Precision parameters of the proposed fiber.

Compound	R.S.D% (single fiber)	R.S.D% (fiber to fiber)
Benzene	1.73	4.28
Toluene	1.86	6.74
Ethylbenzene	3.24	8.62
Xylene	4.83	10.43

### 3.4. Human Hemoglobin-Benzene interaction study using proposed fiber

**Figure 7:**
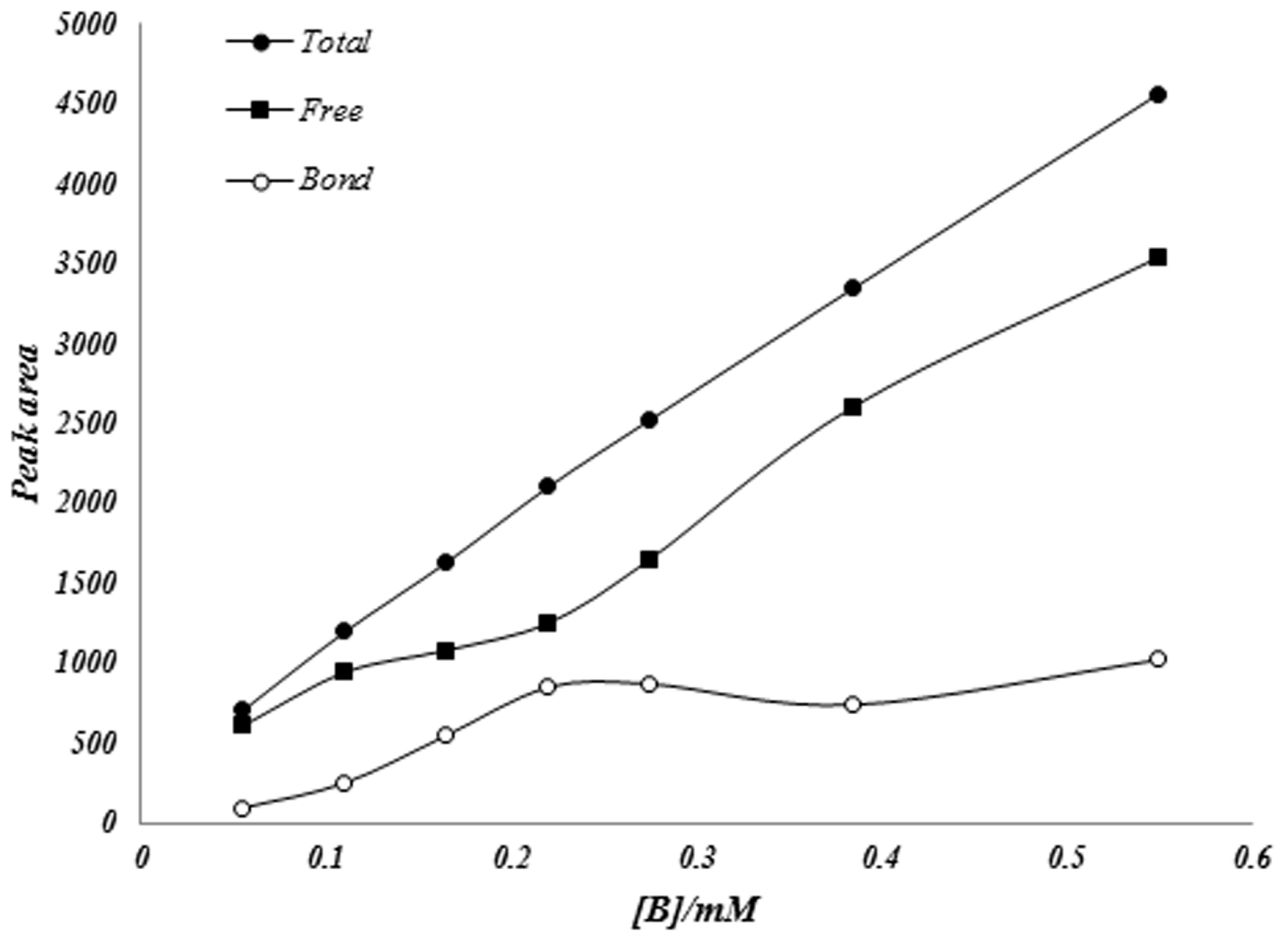
The variation of Area below peak vs. benzene concentration, at 25°C and pH 7.4 in the absence and presence of hemoglobin.

Widespread increasing of poly aromatic hydrocarbons, especially benzene induced several new diseases as mentioned previously. According to this fact that cardiovascular system is one of the first body systems contacts with pollutants, so study of the effect of pollutant on hemoglobin is very important and necessary. So in here we study the effect of benzene on human hemoglobin. Figure 7 shows the plot of peak area versus benzene concentration, in the absence and presence of human hemoglobin (hHb) at specified experimental conditions.

It is obvious that in the absence of Hb, peak area is directly proportional to benzene concentration with linear relationship (R = 0.998). In the presence of hemoglobin the relation between peaks area and concentration is not linear and swerve of calibration curve. This deviation is due to this fact that in the presence of hemoglobin the free concentration of analyte decreased that is due to interaction of analyte with hemoglobin.

In the other hands analyte monomers interact with hemoglobin molecules and so free monomer form of analyte decreased in solution and caused the deviation of calibration curve related to decreasing peak areas corresponding to the free analyte concentrations.

Based on the fact that free concentration of analyte and consequently related peak areas is reduced in the presence of hemoglobin that is due to the interaction of hemoglobin with analyte, the amounts of analyte bond to macromolecule can be calculated. By assuming that saturation of hemoglobin with analyte has been reached when no difference between free analyte concentration in the reference solution and sample solution was observed.

So we can calculate the saturation parameter, Y, in the Hill equation using equation 1 and estimation of saturation parameter [29]:
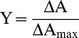
(3.1)


ΔA is the difference of obtained peak areas corresponds to the same added concentration of analyte in the absence and presence of hemoglobin at experimental measurements. So ΔA_max_ is the difference of peaks area at saturation concentrations of analyte that the difference remains constant.

The average number of analyte molecules bound per hemoglobin molecule, ν, has been calculated as [30]: 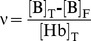
(3.2)


Where [B]_T_, [B]_F_ and [Hb]_T_ are total and free concentration of analyte and total concentration of hemoglobin (Hb), respectively.

**Figure 8:**
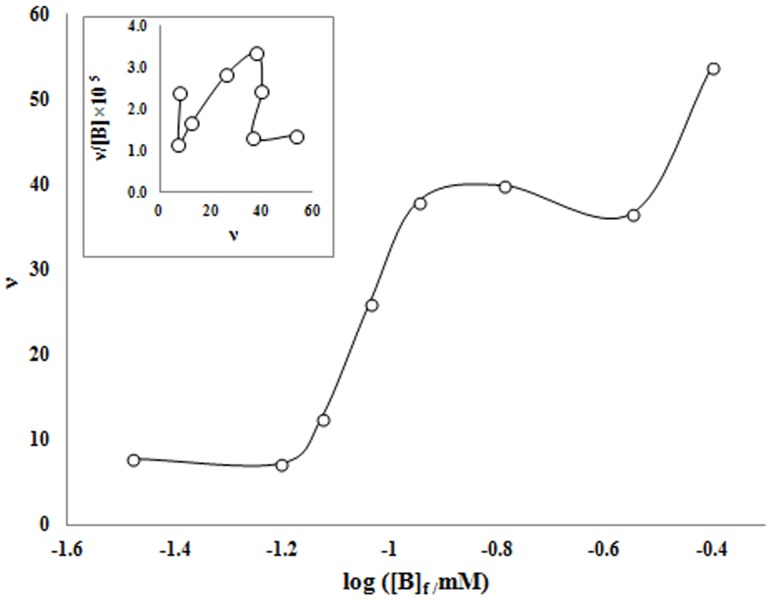
Binding isotherm of benzene interaction with human hemoglobin at 25°C and pH 7.4 (ν define as: average number of benzene molecules bond per hemoglobin) Inset (Scatchard plots for interaction of benzene with human hemoglobin at 25°C and pH 7.4.

Figure 8 show the variation of *ν*, against of the log [B]_F_ (binding isotherm). The corresponding Scatchard plots, (*ν*/ [B]_F_ versus *ν*) and Klotz logarithmic plots are presented in Figure 8 (inset) and 9.

**Figure 9:**
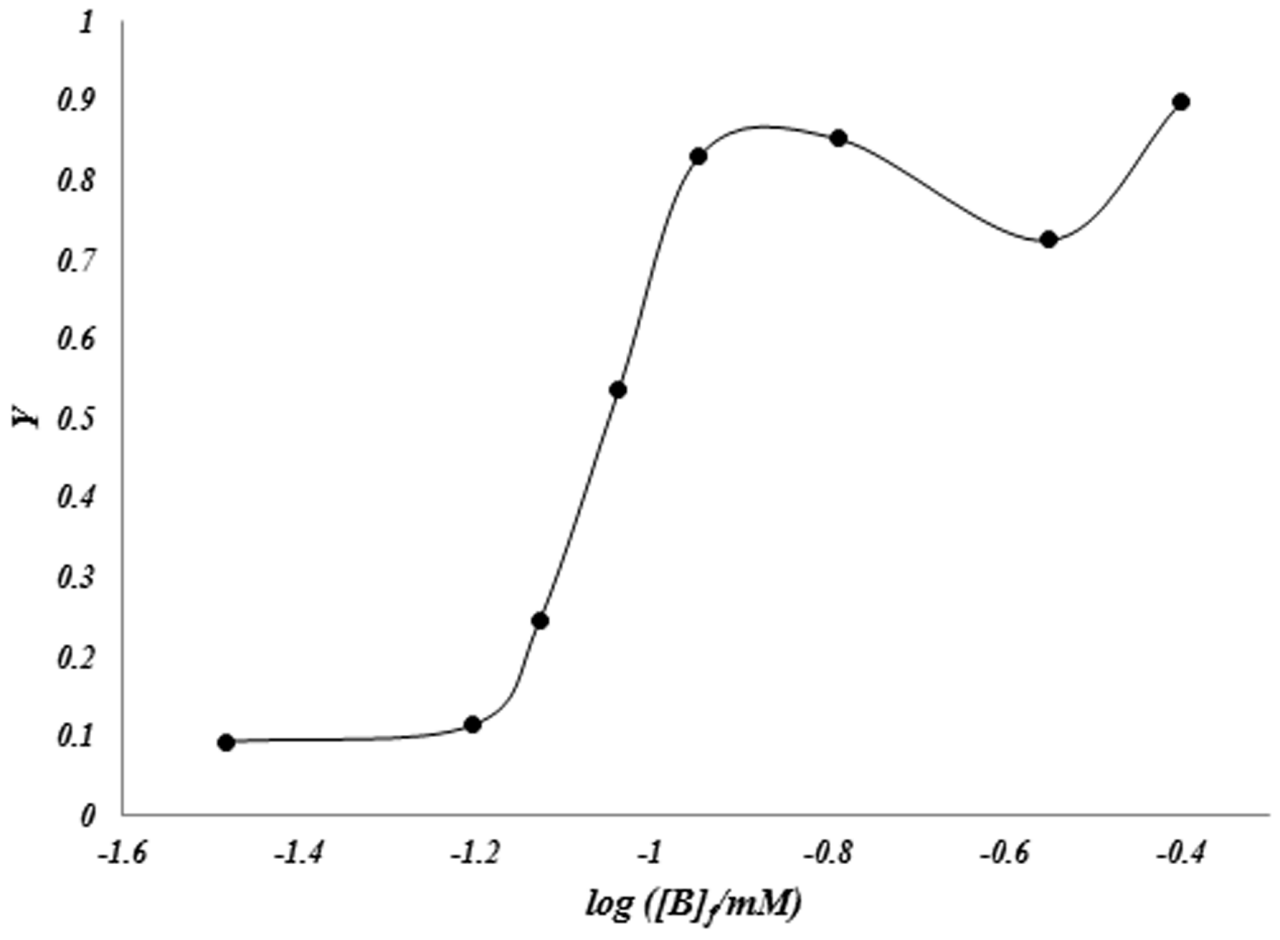
Klotz logarithmic plot for interaction of benzene with human hemoglobin at 25°C and pH 7.4.

The Scatchard plots are unusual and semi-conical). The results used to analyze the binding data based on Hill equation (eq. 3) [30] using estimated saturation factor (eq. 1).
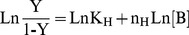
(3.3)


By fitting of data to Hill equation and we can calculate Hill equation parameters. The results are summarized in Table 1.

According to the results it can be seen that benzene bond to hemoglobin in a positive-cooperative (n_H_^>>^1) process. In the half of saturation percent and from Klotz or Hill plot it can be seen that Hill constant is in the molecular interaction rang. Benzene is a hydrophobic molecule and is slightly water soluble, in the presence of hemoglobin, benzene interact with hydrophobic section of hemoglobin for decreasing of water repulsion (hydrophobic forces) and due to this fact that benzene is a small molecule so it can be penetrate to the hydrophobic packet of the molecule and interference on oxygen binding sites. As mentioned in first section of the paper, hemoglobin shows the oxidizing activity in the presence of benzene. By products of this effect maybe have harmful damages on the hemoglobin and other macromolecules in the body. Due to the obtained results, benzene has a good affinity in binding to the hemoglobin molecules. The amounts of Hill coefficient and Hill constant demonstrated that benzene make semi stable complex with hemoglobin. So, by oxidative activity of hemoglobin some free radicals can be produced and radical chain reaction can react with benzene molecules (as a suitable target for the free radicals) [4]. Secondary produced active compound (such as benzoxide and phenol) are strength oxidative compounds that are present on hemoglobin molecules and can make oxidative damages on the macromolecule.

As we know aromatic hydrocarbons are volatile compounds work with these compounds are difficult due to the volatility. Simple spectrophotometric methods are not very suitable methods for these studies due to the limits of detection and sensitivity of methods. Interference of the benzene photometric responses is the other limit in these studies. According to the mentioned points in above, using the accurate, sensitive, simple and suitable procedure in the binding studies of volatile aromatic hydrocarbons are important for obtaining accurate data. In this paper we introduced new SPME fiber constructed from simply synthesized nanocrystaline MMO materials for GC-SPME studies of biological interactions of aromatic hydrocarbon and similar volatile compounds with high sensitivity and accuracy in comparison with spectroscopic methods.

## Conclusion

The study describes a novel SPME fiber based on nanocrystalline mixed metal oxides (as solid sorbent) in polymeric matrix (PVC) coated on a copper wire for BTEX compounds extraction prior to capillary gas chromatographic analysis, and reports its application in binding studies of benzene with human hemoglobin. The proposed fiber has various advantages such as high stability and durability, simple and fast preparation, high sensitivity, high thermal stability, good dispersion and uniform fiber coating. The binding data was accurately obtained using the fiber and decreases the experimental and repeatability errors due to volatility of aromatic hydrocarbons. This method needs very small amounts of hazardous analytes and is very sensitive that is suitable for considering low concentrations of analytes (especially volatile compounds) in interaction studies.
